# Habitat overlap among native and introduced cold-water fishes in the Himalayas

**DOI:** 10.1038/s41598-023-41778-y

**Published:** 2023-09-12

**Authors:** Arif Jan, Ivan Arismendi, Guillermo Giannico, Rebecca Flitcroft

**Affiliations:** 1 Department of Fisheries, Wildlife, and Conservation Sciences, Oregon State University, Nash Hall 104, Corvallis, OR 97331 USA; 2 USDA Forest Service, Pacific Northwest Research Station, Jefferson Way 3200 SW, Corvallis, OR 97331 USA

**Keywords:** Biodiversity, Biogeography, Conservation biology, Ecological modelling, Freshwater ecology, Invasive species

## Abstract

Fish invasions threaten native freshwater ecosystems worldwide, yet methods to map biodiversity in data-deficient regions are scarce. Rainbow trout (*Oncorhynchus mykiss*) and brown trout (*Salmo trutta fario*) have been introduced to the Himalayan ecoregion where they are sympatric with vulnerable native snow trout *Schizothorax plagiostomus* and *Schizothorax richardsonii*. We aim to evaluate potential habitat overlap among snow trout and non-native trout in the Indus and Ganges River basins, Himalayan ecoregion. We transferred maximum entropy (MaxEnt) models developed with spatially continuous freshwater-specific environmental variables to map the distribution of potentially suitable habitats for rainbow and brown trout in the Himalayas. We adopted a similar procedure to map suitable habitats for snow trout species. There were substantial habitat overlaps (up to 96%) among snow trout and non-native trout. Yet, the physiography of receiving basins could play a role minimizing the impacts of each non-native trout on native snow trout*.* We generate high-resolution classified stream suitability maps as decision support tools to help managers in habitat allocation and policy formation to balance recreational fisheries with conservation of snow trout. Our workflow can be transferred to other basins and species for mapping freshwater biodiversity patterns in species-rich yet data-poor regions of the world.

## Introduction

Introduction of fishes has placed freshwater ecosystems among those most affected by biological invasions worldwide^1^. Non-native fishes can modify recipient ecosystems, thereby negatively impacting the diversity and distribution of native fishes^2^. Salmonids have been introduced globally for recreational and commercial purposes, with little regard to their effects on native species^3^. Globally, rainbow trout (*Oncorhynchus mykiss*, Walbaum, 1792), and brown trout (*Salmo trutta fario*, Linnaeus, 1758), are the two most problematic invasive salmonids^4^. The high adaptability of these species outside of their native ranges makes them top ranked in the IUCN’s (International Union for the Conservation of Nature) worst invasive species list^5^. Negative effects of introduced trout species have been documented in many regions including Japan^6^, New Zealand^7^, Chile^8^, Pakistan^9^, and India^10^. Many remote mountainous regions with pristine freshwaters are yet to be studied, including the Himalayas.

Systematic conservation planning in developing countries is difficult to achieve due to limited understanding of freshwater ecosystem functioning, paucity of baseline research, limited professional infrastructure, and inadequate investment in research and monitoring^11^. In the Himalayas, there is a lack of baseline knowledge about freshwater ecosystems including primary biodiversity whereas datasets about suitable habitats at regional scale have been overlooked. Under such data-poor settings, ecological niche models (ENMs) can play a crucial role in providing the best available information on potential distributions based on local and global geospatial information^12^. Though the entire invasion process is complex and multifaceted^8^, ENMs follow ecological theory which suggest that abiotic conditions in the native range of species can be used to predict potential distribution in their introduced range^13^. ENMs are routinely used as assessment tools to anticipate, and prevent the establishment and spread of non-native species^14^.

Unfortunately, datasets and tools needed to implement ENMs in freshwater systems are still limited, especially in understudied regions of the world such as South America, Africa, and Asia. These regions, despite having richest freshwater biodiversity worldwide^15^, are data-poor in terms of primary biodiversity information. High resolution instream and topographic variables are available only for some regions including North America, Europe, and New Zealand^16^. However, high resolution digital elevation models (DEM), which are freely available for most parts of the world, can be used to extract instream and topographic variables. This could be a computationally intensive process, depending on the resolution of DEM. Efficient GIS tools are therefore required to extract spatially continuous topographic variables that better represent stream conditions. These variables can describe the intrinsic potential of habitats^17^ that differentially support both native and non-native species^18, 19^, and be used as covariates to implement ENMs in data poor regions.

In the Himalayas, patterns of occurrence of native and introduced species across riverscapes have been understudied. This ecoregion support high diversity and endemism, with approximately 17% of all the freshwater fishes inhabiting cold waters^20^. Currently, the region faces challenges for the conservation of freshwaters due to pollution, overfishing, glacial retreat, flow regulation, climate change, and non-native species^21^. These factors affect native coldwater cyprinids of the genus *Schizothorax*, commonly known as ‘snow trout’. Although taxonomically misleading, the name ‘snow trout’ is likely attributed to their freshwater residence and similar ecological requirements than trout and other salmonids^22^. Two native snow trout *S. plagiostomus* (Heckel, 1838) and *S. richardsonii* (Gray, 1832) are listed as *vulnerable* on the IUCN red list^9, 23^ due to commercial and recreational fishing pressure. In addition, a considerable truncation and range shift has been observed for these snow trout species attributed to both climate change and non-native rainbow and brown trout^24^. Nonetheless, the geographic distribution of potential habitat overlap between native snow trout and non-native trout species warrants further scrutiny.

Native snow trout and non-native trout thrive in cold, clear waters in high-elevation lakes, streams, and rivers. Both taxonomic groups have produced species complexes independently via convergent evolution with similar ecological roles and requirements in their native ranges^22, 25^. In invaded rivers, naturalized trout populations occupy different habitats and gradients^26^, with rainbow trout using higher elevations^27^ whereas brown trout prefer lower portions of catchments^28^. Here, we use maximum entropy (MaxEnt) models^29^ with attributes of stream networks to evaluate whether introduced non-native trout species would establish differentially across river habitats in the Himalayas. This region has higher mountains and steep elevation gradients, compared to other mountain ranges in the world where these non-native trout have been introduced. We hypothesized (1) a substantial overlap in the distribution of potentially suitable rivers among native snow trout and non-native trout, and (2) a differential overlap pattern between each non-native trout and native snow trout (i.e., more overlap between native snow trout and brown trout at lower elevations, more overlap between native snow trout and rainbow trout at higher elevations).

Our study (1) provides baseline information on species-specific suitable river habitats for native and introduced cold-water species, (2) quantifies the degree of overlap in suitable river habitats among species, and (3) develops a complete workflow for implementing MaxEnt models with ecologically relevant and spatially continuous variables in stream networks. Our findings demonstrate how freely available climatic, landcover, and remotely-sensed topographic variables can be used to create ENMs creating a tool that adds biogeographic realism to the conservation of freshwaters in data-deficient regions of the world. Collectively, understanding the patterns of suitable habitats for native and non-native species and potential habitat overlaps are critical to anticipate and prevent invasions, and to balance the provision of recreational fisheries with the conservation of native species in the Himalayas and elsewhere.

## Material and methods

**Figure 1:**
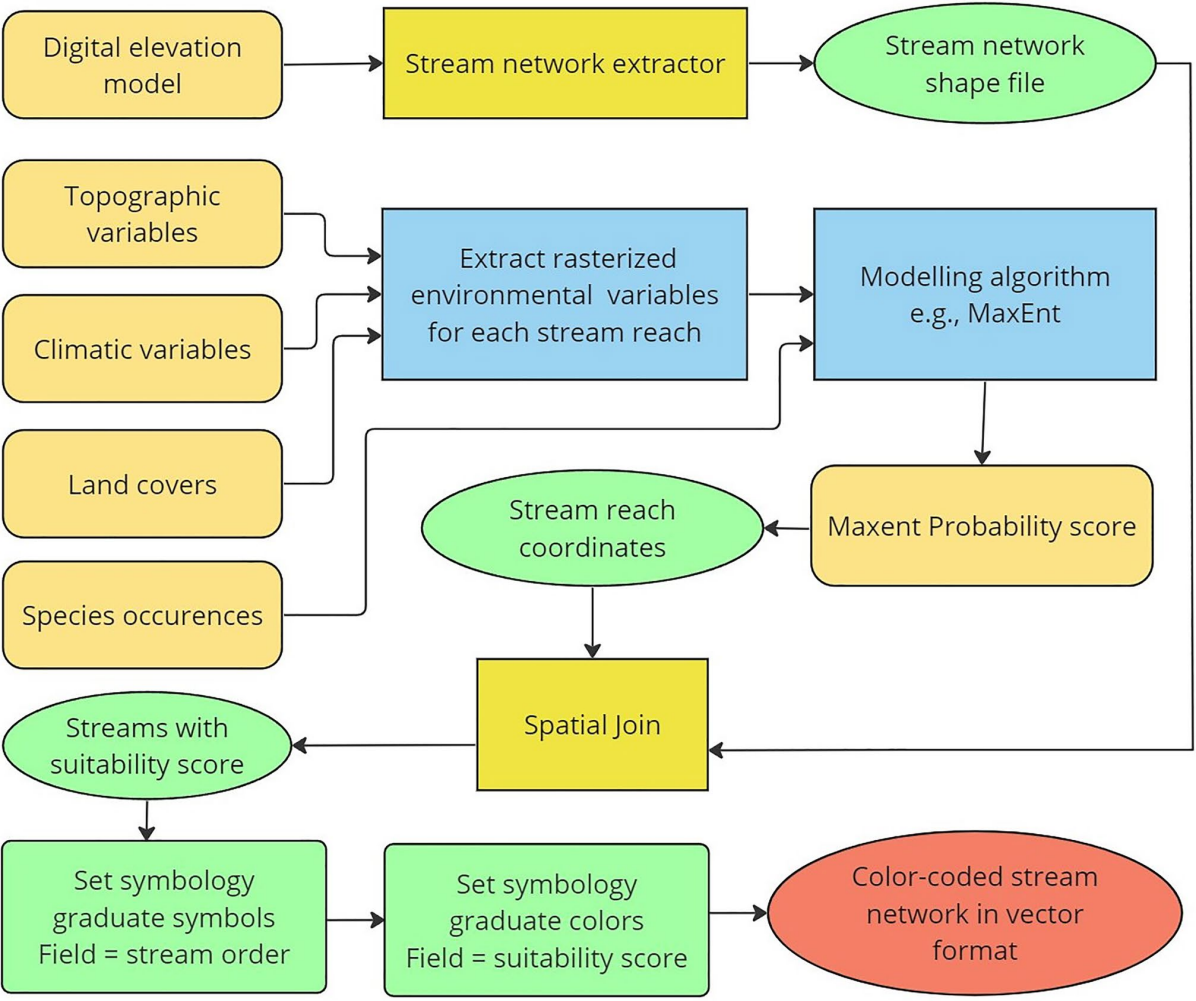
Geoprocessing workflow to extract and display classified stream networks with suitability scores as attribute table in ArcGIS Pro. The brown colored round-edged rectangles represent spatial data as inputs for different stages of the workflow. The yellow rectangles and green ellipses represent geoprocessing tools and their outputs, respectively. Blue rectangles show part of the workflow outside ArcGIS Pro (in R, Python, and other standalone programs e.g., MaxEnt GUI). The green rectangles are different steps in setting symbology (color-coding) for displaying final output. The red rectangles represent outputs that are being used as inputs, whereas the red ellipse represents the classified streams.

We designed a geoprocessing workflow (Fig. 1) in the form of a toolbox that can be used to extract stream networks with associated topographic attributes in data-deficient regions of the world. The topographic attributes of stream reaches can then be compiled to climatic and landcover variables to model the distribution of freshwater taxa. The integrated set of variables represents ecologically relevant predictors for freshwater species distribution which helps in developing more robust ENMs, using different algorithms. Here, we adopted a maximum entropy (MaxEnt) modelling approach^29^ using species occurrence data from both native and introduced ranges, and then the resulting algorithms were applied to the Himalayan basins for predicting habitat suitability for native and non-native trout. We used independently collected species occurrences and experts’ knowledge to evaluate the performance of selected algorithms of habitat suitability.

### Extracting spatially continuous topographic variables: the stream network extractor (SNE)

We developed a complete and customizable workflow as a geoprocessing tool. Stream Network Extractor (SNE) can be downloaded (please see data availability section) and used in ArcGIS Pro for extracting stream networks from DEMs (Fig. 1). Some of the advantages of SNE over existing alternatives are that it allows users to choose the length of stream reach (grain size), and minimum catchment area threshold for delimiting rivers. The order of geoprocessing tools compiled in SNE also helps to reduce computational timing significantly than running all geoprocessing routines separately. SNE can be used in data-deficient parts of the world to extract stream variables from freely available DEM. These reach-scale, spatially continuous stream segments are extracted in vector format, which preserves the hierarchical linear structure of stream networks. In addition to climatic requirements of species, topographic variables derived from DEM add information to the model about the intrinsic potential of streams^17, 30^ to support different fish species^18, 19^. SNE can be used to extract critical habitat features along the hierarchy of the stream network including reach gradient, total upstream gradient, and stream order, which are often associated with hydrological features, and are important for shaping the distribution of fishes^31^. Other instream variables that can be extracted via SNE such as stream density, total upstream catchment area, sinuosity, and density of confluences (stream nodes). These variables are good proxies for capturing habitat characteristics including stream complexity and heterogeneity, which are important drivers of fish distributions^32, 33^.

### Delineation of stream networks

**Figure 2:**
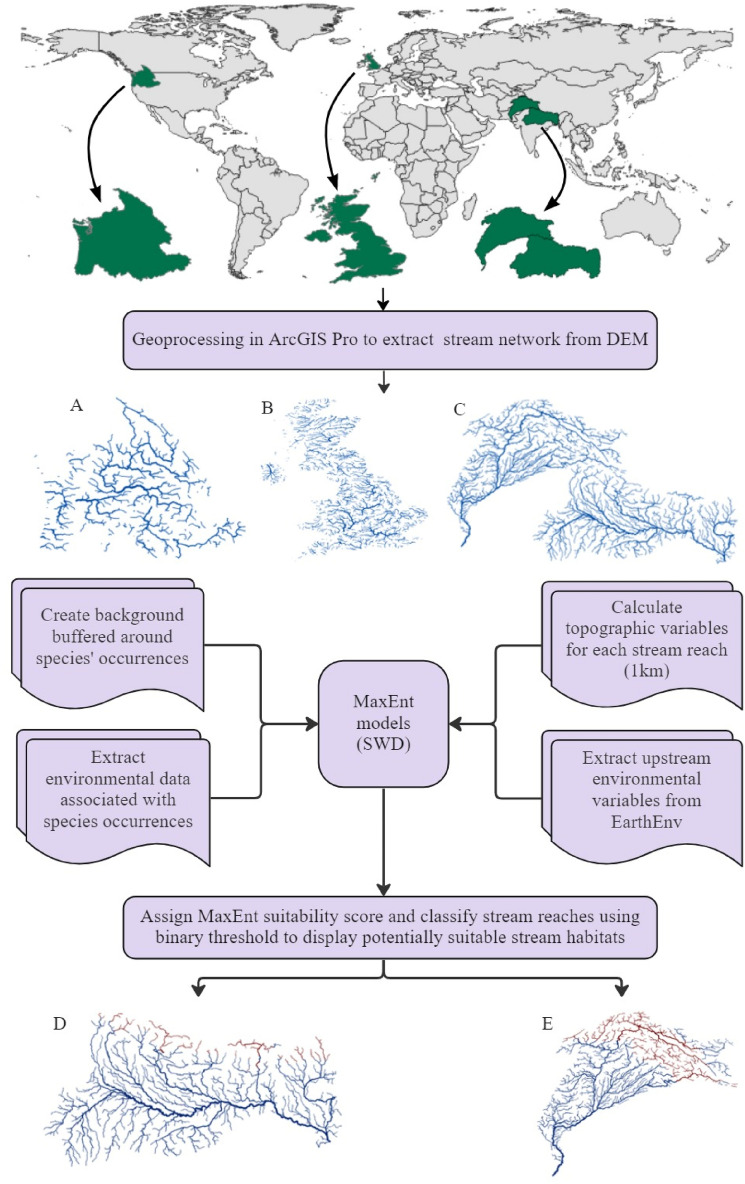
Workflow diagram of data and processes used to develop MaxEnt models of suitable habitats for native and non-native fishes. Stream networks were extracted in ArcGIS Pro, MaxEnt models were built in R, and outputs were visualized back in ArcGIS Pro using appropriate binarization threshold. The geographic regions on the top shows regions of calibration (A-Columbia Basin for rainbow trout, B-United Kingdom for brown trout, C-Himalayan range for snow trout species), whereas stream network at the bottom represent receiving basins (D-Ganges River basin, E-Indus River basin). The figure was produced with ArcGIS Pro 3.0.0 with extensions provided by Oregon State University (https://www.esri.com/en-us/arcgis/products/arcgis-pro/overview).

We used 12.5 m resolution L-Band DEMs (ALOS PALSAR) for stream network extraction; the best available space-borne topographic data for hydrological modeling^34^. Given that populations and communities of stream fishes generally carry out important aspects of their life histories at intermediate spatial scales^35^, we split the seamless stream network (Fig. 2) into 1.0 km stream reaches. We used 2.0 km^2^ as the starting threshold for delimiting headwater streams (i.e., a headwater stream should have, at least, a catchment area of 2 km^2^). We used SNE for extracting the stream network with associated topographic variables in our study area. We choose to extract our own stream network over already available datasets with environmental attributes e.g., HydroSHEDS^36^. HydroSHEDS has a coarser resolution of DEM (~ 450 m) from which the stream network is derived, and larger threshold to delineate headwater streams limits our ability to assess suitable habitats within smaller basins (< 10 km^2^).

### Environmental variables

In stream networks, climate, geology, and topography at large scales set the context for geomorphic processes that create and maintain habitat at finer scales^37^. Freshwater ecosystems are also shaped by hydrological processes occurring upstream and therefore, we used hydroclimatic and landcover information summarized over the upstream catchment area^38^ (Table 1). In addition to rasterized hydroclimatic variables, which regulate fish distribution at large biogeographic scales^39^, topographic variables are associated with fluvial geomorphology of streams^40^, shaping fish distribution at local habitat scale. Landcover and soil organic carbon from EarthEvn^38^ serve proxies for stream productivity^41^, and are important in limiting fish distributions.

**Table 1:** Hydroclimate, topography, landcover and soil predictors of suitable habitats for target species used in MaxEnt models.

Class	Variable	Native snow trout	Non-native brown trout	Non-native rainbow trout
Hydroclimate	Annual mean temperature (°C)^a^	×	×	×
	Temperature seasonality (°C)^a^			×
	Annual upstream precipitation (mm)^a^	×	×	×
	Precipitation seasonality (mm)^a^	×	×	×
Stream network, and topography	Density of stream confluences (Nodes/km^2^)	×	×	×
	Strahler stream order (Unitless)	×	×	×
	Stream density (Km of stream/km^2^)	×	×	×
	Reach gradient (% slope)	×	×	×
	Sinuosity index (Unitless)	×	×	×
	Catchment area (km^2^)^a^	×	×	×
	Average slope (° * 100)^a^	×	×	×
Land cover	Deciduous needleleaf trees (%)^a^	×	×	×
	Evergreen broadleaf trees (%)^a^	×	×	×
	Mixed/other trees (%)^a^	×	×	×
	Shrubs (%)^a^	×	×	×
	Herbaceous vegetation (%)^a^		×	×
Soil	Average soil carbon (%)^a^			×

^a^Measured as the upstream catchment area.

We started with a variety of hydrologic, topographic, and climatic variables, some of which were highly correlated with each other (Supplementary Figures S8–13). Variable selection was guided by ecological relevance, correlation coefficient, data availability, and explanatory power based on iterative model runs. We selected different sets of variables for each species based on the correlation among variables in their native range for snow trout species, and pooled across native and introduced ranges for non-native trout. Only those variables having Pearson’s correlation coefficient < 0.7 were retained^42^ (Table 1). Among the correlated variables, we dropped those that had limited availability in the Himalayas (e.g., we selected upstream catchment area rather than discharge/flow). Detailed hydrological data is limited in most parts the world^43^. We applied the same criterion for climatic and topographic variables when they were correlated with hydrological variables.

### Occurrence data for native and non-native trout

We used presence-background data for MaxEnt model development. Absence data is not informative in data-deficient settings where sampling sites are not thoroughly surveyed, and where sampling methods and intensities are inconsistent^12^. This is often the case in developing countries due to limited resources for research^44^. We contrasted occurrence data with pooled, random, background points (stream reaches), carefully selected inside a buffer around occurrences (see details in Supplementary Fig. S3).

In the native range of rainbow trout, we compiled occurrences (n = 1801) from the Oregon Department of Fish and Wildlife, and Pacific States Marine Fisheries Commission. In the native range of brown trout, we obtained occurrences (n = 2279) from National Biodiversity Network United Kingdom. In the Himalayas, we obtained occurrences of snow trout (*S. plagiostomus*; n = 255), and non-native trout (n = 98 for rainbow trout; n = 82 for brown trout) by conducted field sampling using cast and scoop nets between January 2017 and September 2019. We only recorded the coordinates of fish presence, i.e., the caught fishes were released back immediately to their natural habitat. Our involvement with fish was least invasive and according to the guidelines of the fisheries department of Khyber Pakhtunkhwa, Pakistan and local government authorities. Lastly, we extracted occurrence data for snow trout (*S. richardsonii*; n = 244) from published literature^45^, and the Global Biodiversity Information Facility. We filtered all occurrences for potential errors associated to unknown/assumed datum, duplicates, and fuzzy references. We discarded occurrences with geographic uncertainty > 100 m.

### Model development and transfer

We used maximum entropy model (MaxEnt) version 3.4.4^46^ for all four species. MaxEnt has been widely used for presence-only data producing consistently competitive and ecologically meaningful predictions^47^. We transferred MaxEnt models from a significant portion of the native ranges of rainbow trout (Columbia River Basin; 440,718 km of streams), and brown trout (United Kingdom; 234,867 km), to predict suitable habitats in the Indus (147,541 km), and Ganges (36,457 km) Basins from the Himalayan (Fig. 2). The portion of native ranges where the models were developed exhibited sufficient heterogeneity to capture the environmental limits of non-native trout, as evidenced by the generation of Gaussian response curves for the employed environmental variables. We adopted a similar procedure to map suitable habitats for native snow trout species in these two Himalayan basins. We used Kuenm R package^48^ to fit MaxEnt models. This R package allowed for comparisons in MaxEnt among candidate models under different regularization multipliers, and feature classes, balancing predictive power with appropriate complexity and statistical significance. Candidate models were evaluated for statistical significance (partial ROC), omission rate (E) and model complexity (AICc), to select the best model (see Supplementary Figs. S4–7). Given the good quality of occurrence data for *S. plagiostomus* and non-native trout, we used “minimum training presence” threshold to binarize MaxEnt probabilities. For *S. richardsonii*, since part of the data comes from GBIF, we used “10 percentile training presence” threshold, which leaves a 10% margin of error in the occurrence records and assumes that 10% of occurrence records in the least suitable habitat are not occurring in regions that are representative of the species overall habitat, and thus should be omitted.

### Model evaluation

#### Evaluation using independent data

We evaluated our final models using independent datasets. Testing on an independent dataset has often been considered the most robust type of evaluation^49^. The final MaxEnt models (average of 10 folds cross validation) for non-native trout had omission rates of 1–2% on validation data. Omission rates on independent data increased to 7–18%, which is to be expected for cross-continental model transfer^12^. Unfortunately, we could not conduct any sampling to collect independent data for *S. richardsonii*. Using Receiver Operating Characteristic (ROC) analysis for model evaluation has been criticized for giving equal weight to omission and commission errors^50^. Models for predicting suitable habitats for non-native/invasive species may have less tolerance for omission error than for commission error. Therefore, we used partial ROC (pROC) developed for ENM evaluation^50^. pROC uses AUC ratios (The partial AUC divided by random expectation), where a value of 1.0 represents model performance no better than random, whereas models with AUC ratios near or greater than 2.0 are considered good^51^. The *p* values of pROC indicate whether the ratios of model AUC to the random AUC is statistically significant. The details of evaluation metrics for our final MaxEnt models for each species are provided in the Supplementary Table S1.

#### Evaluation using experts’ opinions

Although our models performed well on independent data, we also included expert opinion in the evaluation process. We used the Delphi method^52^ and conducted Qualtrics surveys requesting respondents to evaluate maps of suitable habitats for our target species. We requested respondents to give a score (between 0 and 10) about the overall accuracy of the maps of Himalayan native fish. In the first round, mapped outputs of our final models were shared with 51 coldwater fisheries experts from Pakistan, India, Nepal, and Bhutan. A total of 16 out of 51 experts responded our survey. We adjusted model parameterization incorporating suggestions from experts before producing our final suitability maps. The final adjusted maps were made available to all the respondents. In the second round, the respondents agreed to the adjustments made and showed confidence in the final adjusted maps. Details of the gridded maps for each species, and questions asked to the experts, are provided in the Supplementary information. We reviewed the decision tree from the Office of Research Integrity and Institutional Review Board and confirm that all methods were carried out in accordance with relevant guidelines and regulations from Oregon State University. Institutional pre-screening indicates that our work is exempted from IACUC and IRB as no animals nor human subjects were used to conduct our research.

## Results

### Species-specific distribution of potentially suitable habitats

**Figure 3:**
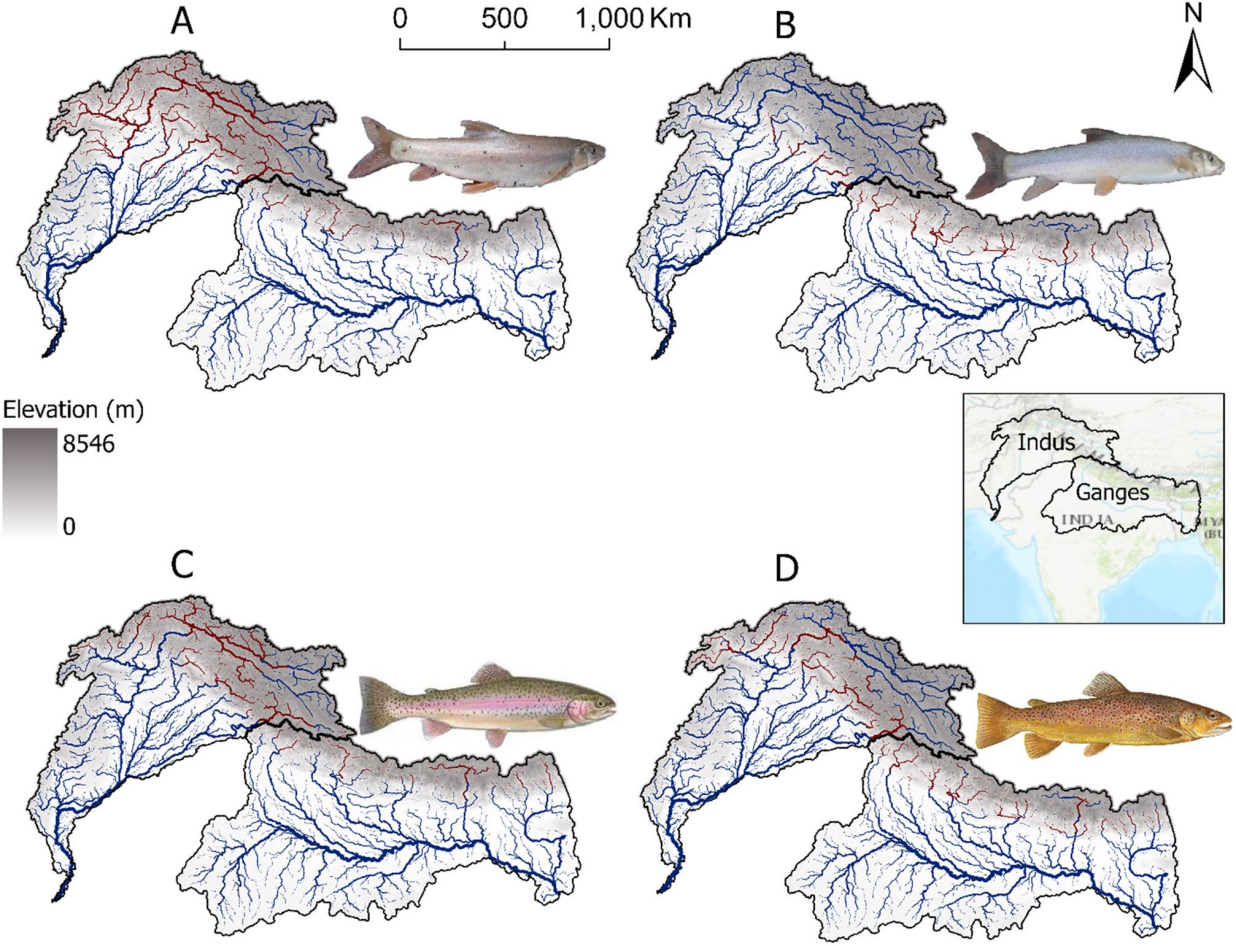
Maps of habitat suitability for native snow trout and non-native trout species in the Indus and Ganges River basins, Himalayas. Dark red represents potentially suitable streams whereas dark blue represents unsuitable streams. Only 4th and higher order streams are shown for better visualization. Map A and B represent potentially suitable habitats for native snow trout *S. plagiostomus* and *S. richardsonii,* respectively. Map C and D represent potentially suitable habitats for non-native rainbow and brown trout, respectively. The figure was produced with ArcGIS Pro 3.0.0 with extensions provided by Oregon State University.

Species-specific distribution maps of potentially suitable habitats (Fig. 3) indicated that the snow trout *S. plagiostomus* had an extended distribution in the Indus (151,974 km) compared to the Ganges (36,457 km) River basin. This contrsated with snow trout *S. richardsonii,* that had more suitable habitats in the Ganges (101,898 km) compared to the Indus (29,177 km) River basin. Although non-native trout have potentially suitable habitats in both Himalayan basins, they were differentially distributed gradient-wise. Suitable habitats for rainbow trout dominated higher elevation areas, whereas brown trout habitats were more abundant at lower elevations. Assigning MaxEnt suitability score as an attribute to the stream network allowed us to quantify the total length of potentially suitable streams for each species. The total length of potentially suitable streams for rainbow trout in the Indus and Ganges Basins were 124,596 km and 13,861 km, respectively. Similarly, the total length of potentially suitable streams for brown trout in the Indus, and Ganges River basins, were 103,701 km, and 62,102 km, respectively. We evaluated the performance of our final maps with experts knowledge. We received a reasonable average score (8.2/10) for our final adjusted distribution maps.

### Habitat overlap between non-native trout and native snow trout

**Figure 4:**
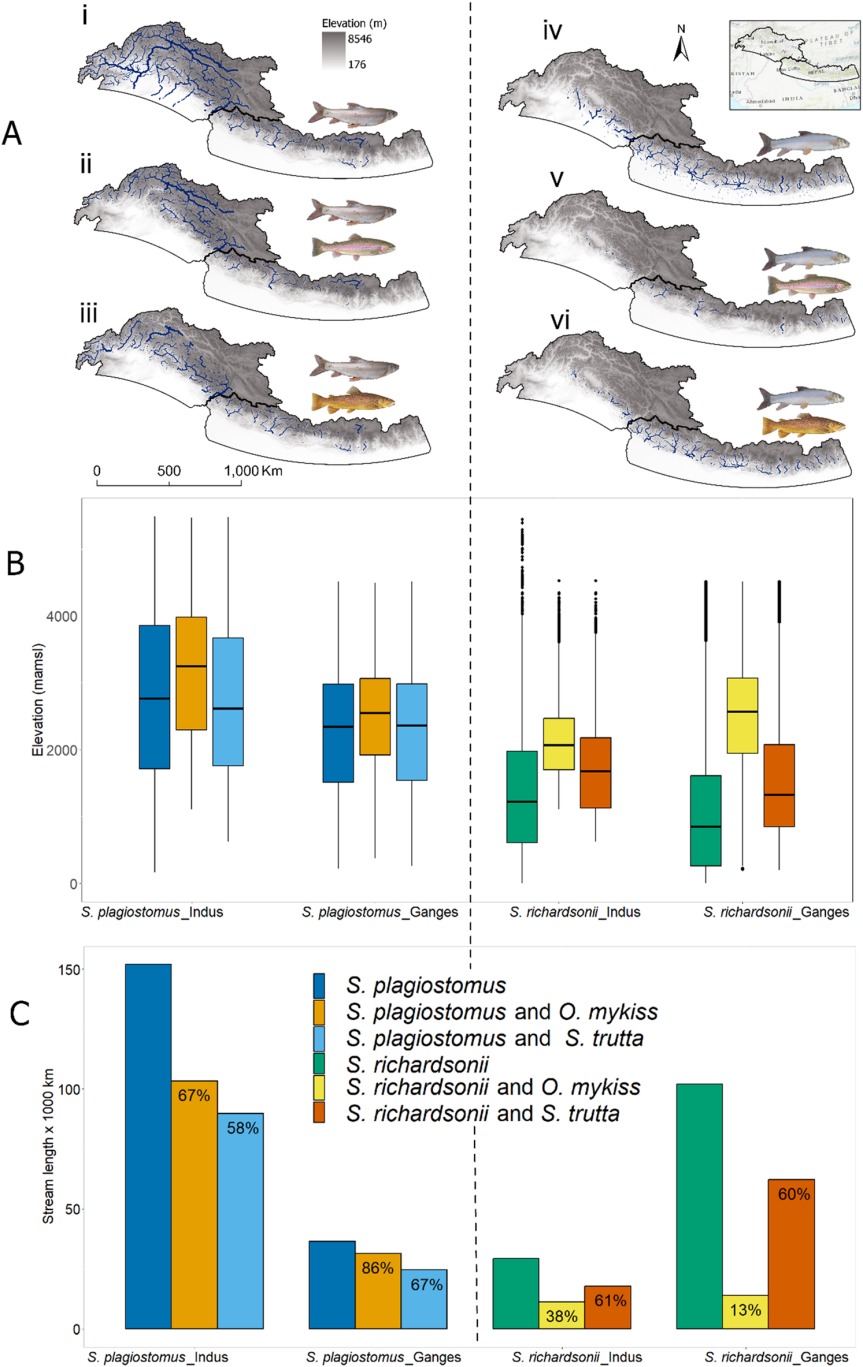
Map of suitable habitats for native snow trout species and the overlap with non-native trout species in the Indus and Ganges Basins. Left-side panels correspond to the native snow trout *S. plagiostomus*, whereas right-side panels refer to the native snow trout *S. richardsonii*. (**A**) Maps i, ii, and iii show the distribution of *S. plagiostomus* and its overlap with rainbow and brown trout, respectively. Similarly, maps iv, v, and vi correspond to *S. richardsonii*. Only 4th and higher order streams are shown to improve visualization. (**B**) Violin plots show the gradient-wise distribution of individual and overlapped habitats. Figures B and C share the same legend. (**C**) Total length (km) of individual and overlapped habitats. The percentages on the bars represent habitat overlaps between paired native snow trout and non-native trout species. The figure was produced with ArcGIS Pro 3.0.0 with extensions provided by Oregon State University.

The highest overlap in suitable habitats between native and non-native trout species occurred for the snow trout *S. plagiostomus* (Fig. 4a). In the Indus River basin, suitable habitats for this snow trout species overlapped with suitable habitats for rainbow trout in 67% of stream reaches, and for brown trout in 58% of stream reaches. Overall, 78% of suitable stream reaches for this native snow trout overlapped with at least one non-native trout. In the Ganges River basin, the overlap of suitable stream reaches was higher, with 86% for rainbow trout and 67% for brown trout. In addition, 96% of suitable habitats for this native snow trout overlapped with at least one non-native trout in the Ganges River basin (Fig. 4c).

In the case of *S. richardsonii*, 38% and 61% of potentially suitable stream reaches overlapped with rainbow and brown trout in the Indus River basin, respectively. In the Ganges River basin, the overlap resulted in 13% and 60%, respectively. Overall, 63% and 62% of suitable stream reaches for this native snow trout overlapped with at least one non-native trout in the Indus and Gages River basins, respectively (Fig. 4c).

As mentioned above, suitable stream reaches for rainbow trout occurred at relatively higher elevations compared to brown trout, in both basins. The wide elevation range of suitable habitats for the native snow trout *S. plagiostomus* (mean elevation = 2784 m.a.m.s.l.) resulted in a higher overlap with both non-native trout species. In contrast, suitable stream reaches for the native snow trout *S. richardsonii* had more restricted elevations (mean elevation = 1078 m.a.m.s.l.), resulting in a higher overlap with brown trout compared with rainbow trout.

Despite the substantial overlap between the distribution of snow trout and non-native trout, the mean elevations at which potentially suitable streams are distributed were statistically significant (Kruskal–Wallis test *p* < 0.01) and different for each native-non-native pair (Fig. 4b). Potentially suitable stream reaches for *O. mykiss* were distributed at significantly (Wilcoxon test *p* < 0.01) higher elevations than suitable stream reaches for *S. trutta fario*. On the other hand, potentially suitable stream reaches for *S. plagiostomus* were distributed over a wide range of elevations. As a result, overlapped in potentially suitable stream reaches between *S. plagiostomus and O. mykiss* were at significantly (Wilcoxon test *p* < 0.01) higher elevations than overlapped streams between *S. plagiostomus* and *S. trutta fario.* Potentially suitable streams for *S. richardsonii* overlap more with *S. trutta fario* compared to *O. mykiss*, as both species inhabit relatively lower elevations (Fig. 3a). Elevation of potentially suitable stream reaches were also significantly different by basin. Overlapped streams between *S. plagiostomus* and non-native trout were at significantly higher elevations in the Indus (2942 m.a.m.s.l.) compared to the Ganges (2347 m.a.m.s.l.) River basin. Similarly, overlapped streams between *S. richardsonii* and non-native trout were at significantly (Wilcoxon test *p* < 0.01) higher elevations in the Indus (1847 m.a.m.s.l.) compared to the Ganges (1690 m.a.m.s.l.) River basins.

## Discussion

Our findings lay out baseline information on reach-level (1 km) potential suitability of streams to support species-specific habitats for native snow trout that can be used to reduce the risk of new introductions, and conserve sensitive habitats in the Himalayas. Our geoprocessing workflow characterizes stream networks to be used in ENMs and is transferable for modelling freshwater species distribution at fine scales (< 1 km). The last is especially relevant in data-deficient, but species-rich parts of the world. MaxEnt models outputs provide native snow trout potential suitability maps on a continuous probabilistic scale and help the identification of conservation areas most suited for this species (Supplementary Fig. S1). Specifically, highly suitable streams for snow trout in the Indus River basin mainly occur in Chitral, Swat, Dir upper, North-eastern Gilgit, with some fragmented segments of the Jhelum River, and the upper parts of the Chenab, Ravi, and Bias Rivers in the Indian territory. Whereas in the Ganges Basin, highly suitable streams for snow trout lie mostly in north-eastern India (Uttarakhand and Himanchal Pradesh), Nepal, and Bhutan (Supplementary Fig. S1). In addition, we provide habitat suitability maps that can be used to inform stocking practices of non-native trout in streams where they could potentially establish naturalized populations.

Species-specific maps provide valuable guidance for prioritizing habitats where native snow trout populations could be protected or restored, but additional knowledge about the life history traits of native species can be included to minimize competition and spatial overlap with non-native fishes^53^. We show that both rainbow and brown trout follow global parallelism resulting in a significant habitat overlap with native snow trout, although with different distributions following elevation as shown in invaded systems elsewhere (e.g. in Japan^6^, New Zealand^7^, and Chile^8^). Rainbow trout tends to inhabit slow-moving, deep-water streams with a 1:1 pool-riffle ratio^54^. Thus, this species might be unable to establish self-sustaining populations at high elevation at northern Himalayan streams where the likelihood of spawning and rearing would be low due to high water velocities. At high elevations, snow trout might have a competitive advantage over rainbow trout due to their specialized morphological traits (e.g., modified lower lip for adhesion), and to preference for torrential streams. Yet, non-native trout invasion would affect *S. plagiostomus* as most suitable for the last are located at lower elevations compared to *S. richardsonii*. Our findings can contribute to the conservation of both snow trout species as the IUCN^9^ recommends the reduction of stocking of non-native trout via hatcheries, and their restriction to stream segments that would minimize any likelihood of naturalization.

Recent research has documented range truncation for native snow trout due to non-native trout introductions^9^ and naturalized populations in some parts of the Ganges^10^ and Indus River basins^55^. However, naturalization to a new stream and climate is a necessary, but not sufficient condition for invasion success^56^. Nonetheless, the growing concerns regarding potential invasiveness^57^ are valid as non-native trout may become invasive, as observed in other basins^10^ under continuous and high propagule pressure. Previous research demonstrated that even sub-optimal climatic conditions allow colonization of invasive species if propagule pressure is sufficiently high^58^. Yet, the lack of consolidated data on propagule pressure may underestimate the risk of non-native trout establishing themselves and ultimately invading the Indus and Ganges River basins in places our models identified as suitable stream reaches. Further research to document the frequency and magnitude of trout stocking areas warrants further attention in this region.

Our results highlight streams vulnerable to non-native trout invasions at the basin scale in the Indus and Ganges River basins. Although at the basin scale climatic conditions are likely the main drivers of fish distribution^59^, non-native trout would experience a series of local scale filters in the form of physiological thermal constraints, biotic interactions, and access to ideal stream habitat with intrinsic potential^17^ before they can establish naturalized populations in a particular stream or watershed. Although we are unable to incorporate local-scale biotic interactions due to a lack of data, our results can identify both potential locations to further study the ecological interactions among native and non-native fishes^10^ and areas where stocking trout might be less detrimental for native species. For instance, rainbow trout stocking at the upper reaches of River Swat may not as ecologically detrimental as in River Kumrat as it has more chances of naturalization in Kumrat watershed^55^. Similarly, Rivers in Chitral watersheds where non-native trout outcompete native snow trout^21^, reduced stocking and management by modifying impacts of non-native trout, as outlined by Dunham et al.^53^ would help in the conservation of native snow trout.

Our approach has some limitations that need to be acknowledged to implement best practices and proper use of ENMs in the management of biological invasions. First, the regulatory role of climatic and abiotic variables in freshwater fish distributions is typically observed at broader biogeographic scales. However, the direct use of air temperature and precipitation as surrogates for actual instream hydrological conditions is tenuous^60^. Stream temperature and hydrology are fundamental determinants of fish distributions^61^, and are highly correlated to air temperature and precipitation. This association is also affected by other factors such as riparian vegetation^62^ total catchment area, hyporheic exchange, slope, and watershed elevation^40^. In addition, the correlation between air and water temperature also becomes weaker over time^60^ and at higher elevation potentially affecting habitat modelling for cold-water fish species^63^. We minimize these potential limitations by testing our final models with independent data from the receiving basins and with expert judgement. The final predicted habitat suitability for all species in this study closely approximated the expert’s knowledge of fish distributions, giving us confidence in the utility of our models for decision support to managers across Himalayan countries.

Additional limitations of our modelling approach include the data deficiency in developing countries as well as the overall assessment of impacts of invasive species in freshwaters. Here, we provide a geoprocessing tool SNE that can be used to extract important topographic stream variables from freely available DEM that can be combined with climatic data and species occurrences to model species distributions. Fortunately, even in situations where occurrence data is absent, experts knowledge about species ecology can be used to model the intrinsic potential of habitat to support fish populations^18, 19^. The SNE tool can extract instream variables (discharge, gradient, and valley confinement) for modelling the intrinsic potential of streams to support species-specific habitat based on ecological knowledge of species requirements. We recommend using DEMs with the finest available resolution to extract more reliable positioning of stream network. With SNE users can run analyses at multiple scales by choosing desired grain size (reach length) and can customize drainage threshold for delimiting headwater streams.

Globally, the Himalayas is one of the most vulnerable regions to climate change. This region is warming at twice the rate of the global average, and glacial retreat is happening six times faster than in other regions of the world^64^. The geographic distribution of native snow trout is likely shrinking, and shifting, under climate change^24^. Many developing countries have extensive freshwater systems with high species diversity and endemism, which demands approaches that maximize the utility of freely available information (e.g., climatic, landcover, and other geospatial data). This study demonstrates that freely available georeferenced species collections from different inventories, DEM, and climatic and environmental data can be used to develop ENMs that provide baseline information for policy making. Therefore, despite the limitations of this study, which are germane to many species distribution studies, it is reasonable and urgent to balance the need for clear baseline research with the reality of limited resources and data deficiency in developing countries.

### Supplementary Information

Supplementary Information.

## Data Availability

All programming code, model outputs, raw data used, and SNE tool were made available for this peer-review process through the open data repository Dryad (https://datadryad.org/stash/share/3U5qi2Xo52W7uPlPA7sRRk9Hgagi1fEvvGFNSdaapr8). Any additional information related to study can be obtained from corresponding author upon reasonable request. Rainbow trout data: https://nrimp.dfw.state.or.us/FHD_FPB_Viewer/index.html. https://maps.psmfc.org/server/rest/services. Brown trout data: https://nbnatlas.org/. *S. richardsonii* data from GBIF: 10.15468/dl.m3xxse.
