# Supplementary material for: Habitat overlap among native and introduced cold-water fishes in the Himalayas
Source: Sci Rep. 2023 Sep 12;13:15033. doi: 10.1038/s41598-023-41778-y (PMC10497582; doi:10.1038/s41598-023-41778-y)
Supplement: Supplementary file 1 — Supplementary Information. [file 41598_2023_41778_MOESM1_ESM.docx]

**Supplementary information**

1. **For local watershed scale conservation priorities, here we present the probabilistic map from MaxEnt output for native snow trout species only.**


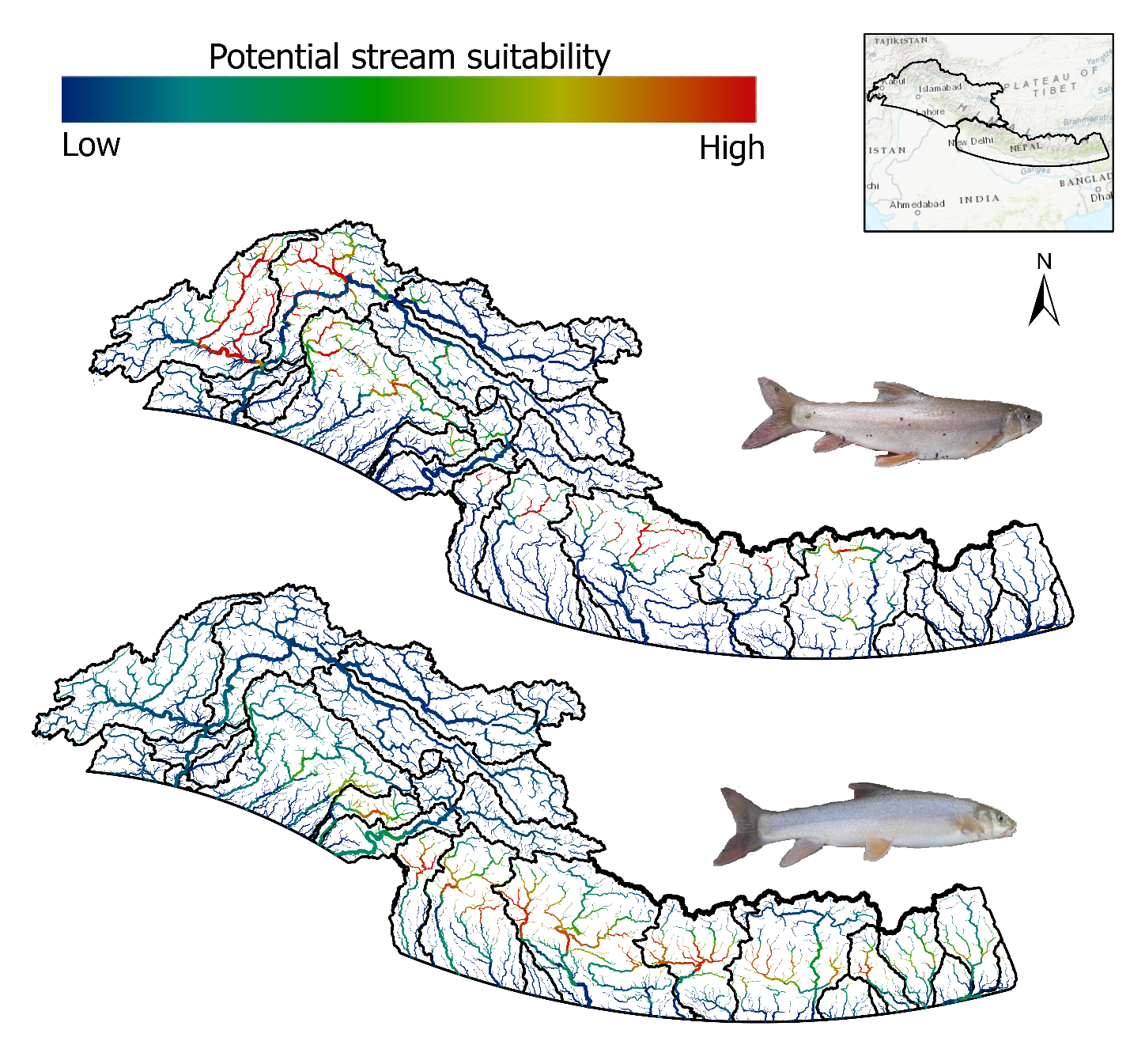


**Figure S1.** Potentially suitable stream habitats for native snow trout on probabilistic scale. Warmer color represents potentially suitable streams. The upper map is for *S. plagiostomus* whereas the lower map is for *S. richardsonii*. The figure was produced with ArcGIS Pro 3.0.0 with extensions provided by Oregon State University (<https://www.esri.com/en-us/arcgis/products/arcgis-pro/overview>).

1. **ArcGIS Pro workflow to extract stream network with spatially continuous topographic information**

We developed the Stream Network Extractor (SNE) tool using the model builder in ArcGIS Pro (**Fig. S2**). Boxes represent the geoprocessing tool used (yellow) whereas ovals represent the corresponding outputs (dark green). For details of each tool such as hyperparameter tunning, download the tool here (<https://datadryad.org/stash/share/3U5qi2Xo52W7uPlPA7sRRk9Hgagi1fEvvGFNSdaapr8>). Once the tool is downloaded, load it to toolboxes in the catalog panel of ArcGIS Pro. Right click on the model builder and click on edit to see and edit the model (Stream Network Extractor) in model builder window.


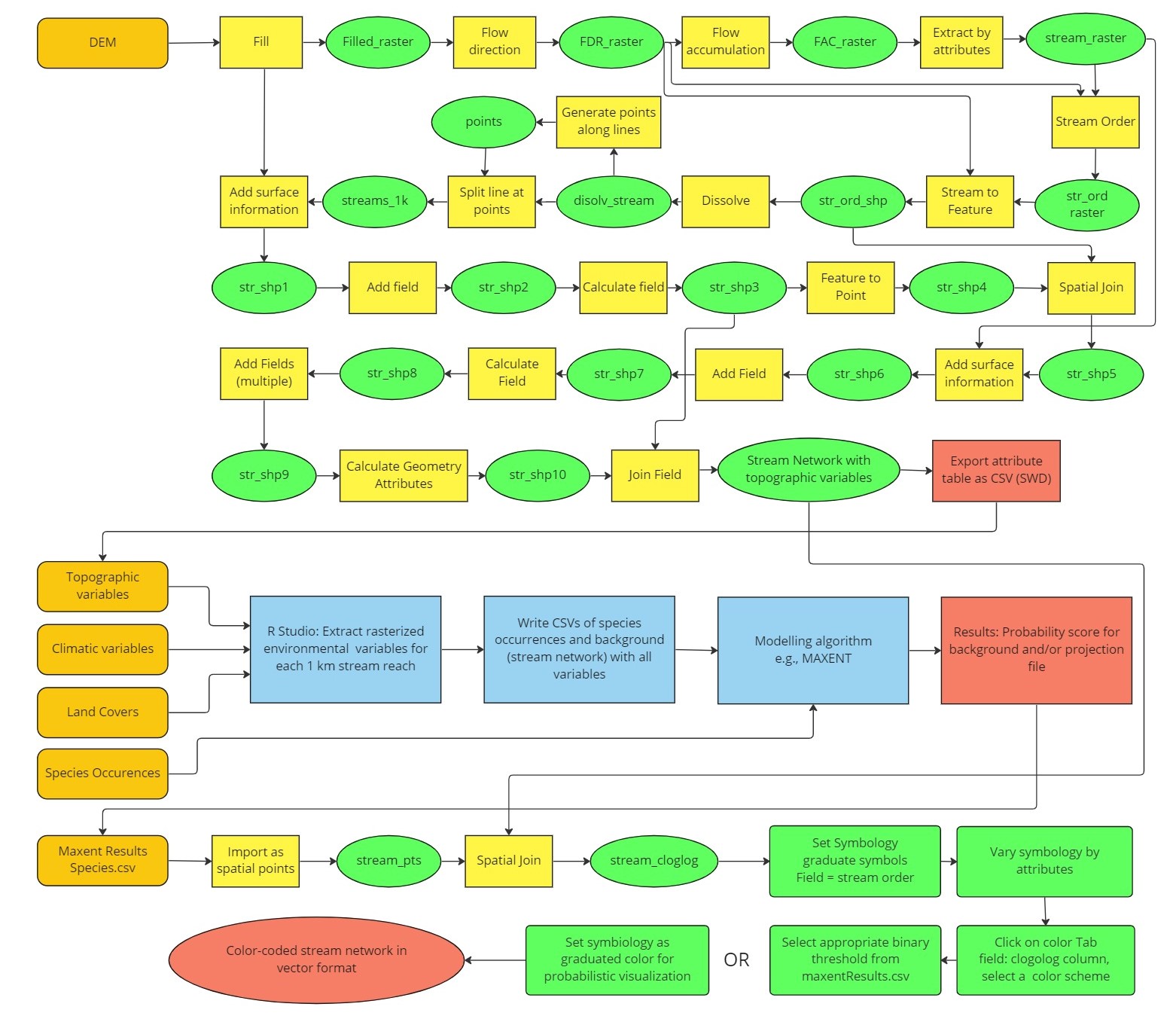


**Figure S2.** Geoprocessing workflow to extract and display classified stream networks with suitability scores as attribute table in ArcGIS Pro. Brown rectangles represent spatial data as inputs for different stages of the workflow. The yellow rectangles and green ovals represent geoprocessing tools and their outputs, respectively. Blue rectangles show part of the workflow outside ArcGIS Pro (in R, Python, and other standalone programs such as MaxEnt GUI). Green rectangles represent different steps in setting symbology (color-coding) for displaying final distribution maps. Red rectangles represent outputs that are being used as inputs, whereas the red oval represents the classified streams. This figure can be downloaded in high resolution from here (<https://datadryad.org/stash/share/3U5qi2Xo52W7uPlPA7sRRk9Hgagi1fEvvGFNSdaapr8>).

1. **MaxEnt background selection**

We calibrated models for rainbow and brown trout using environmental information and occurrence data pooled across native and introduced ranges (i.e., global model). This procedure is appropriate to capture larger spectrum of environmental conditions where the species may exist and spread ^1^. For the two native snow trout (*S. plagiostomus* and *S. richardsonii*), a common single background was used buffered (100 km) around their occurrences. We used bias files for selecting MaxEnt backgrounds using the SDM toolbox Pro version 0.9.1 ^2^ to restrict background selection only within a certain buffer around species’ occurrences. This criteria allowed us to add only those meaningful pseudoabsences to the model that were within the accessible range of the species ^3^. This minimizes the effect of both sampling and latitudinal biases associated with occurrence data, a standard for best practices in studies involving species distribution models (Araújo et al. 2019). To further minimize spatial biases, we thinned occurrences for all four species to one presence per stream reach using spThin package ^4^ in R. Also, we used a point process model to estimate an adequate number of background points ^5^. The convergence plot from this analysis showed that background points converged around 50,000-200,000 (**Fig. S2**). However, the regularized training gain of MaxEnt did not increase beyond 50,000 points. Therefore, to calibrate MaxEnt models we used 50,000 pooled, random, background points (stream reaches) along with species presence points.


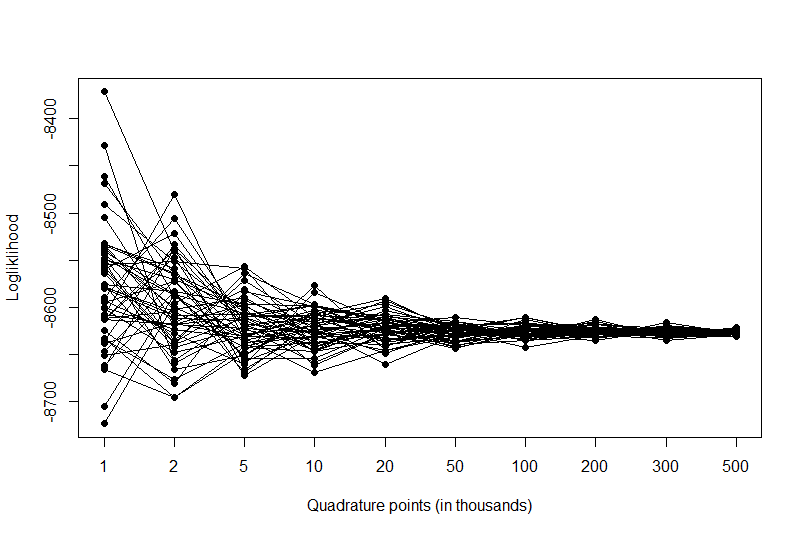
**Figure S3**. Convergence plot for optimal quadrature (background) points vs. log-likelihood. R codes used to create this plot are provided in the supplementary material from Renner et al. (2015).

1. **Displaying classified streams**

Distribution models for terrestrial species take input data as raster and produce results in the form of output raster. However, streams are better represented as vector shape files and attributing stream suitability score to each stream reach is a separate post-model step in a GIS software. The model output (Probability values) for each stream reach is analogous to probability score for each pixel in a raster. Here, we show an example for MaxEnt model. MaxEnt results folder produces a probability score (e.g., cloglog values) in the projection file (if the model is projected in space and/or time). The output CSV file usually will have three columns including the first two as geographic coordinates, and the third column as the probability score (e.g., raw MaxEnt score or cloglog). In our case, we adopted the following steps: 1) Display MaxEnt output file in ArcGIS pro; 2) Display the original stream network file with attributes in the same window as in 1); 3) Both shape files should have the same number of rows (check the ‘attribute table’); 4) Use the ‘join table geoprocessing tool’ to transfer the probability score to the strem network attribute table; 5) Right click on the stream network shape file and go to ‘symbology’; 6) Display the stream network as graduate symbols using the stream order field. This will provide a better visualization of a stream network; 7) Then click on the ‘color tab’ in the symbology and use an appropriate threshold to binarize the stream network. Note that the values of different thresholds for different types of MaxEnt outputs can be found in the “MaxEntResult.csv” file in the output folder, as illustrated in blue rectangles in **Fig. S2**.

1. **Model selection, based on AICc, omission rate and partial Receiver operating characteristic (pROC)**

We used the Kuenm package ^6^ in R to calibrate a number of candidate models that were contrasted and ranked using pROC, omission rate (E), and model complexity (AICc). The Kuenm package allows to compare candidate models under different regularization multipliers, and feature classes in MaxEnt, balancing predictive power with appropriate complexity and statistical significance. Global models were calibrated for non-native trout and then transferred to Indus and Ganges River basins for prediction of habitat suitability. Similarly, native models were developed for snow trout that were interpolated to evaluate suitable habitats in the Indus and Ganges River basins. After examining a series of plots showing the distribution of candidate models along a gradient of AICc values (x-axis) and the omission rate at 1% (y-axis), we used this omission rate threshold. Thus, our model assumed 1% error rate in our occurrence data after considering the quality of our occurrence data. See the illustrative example in **Figs. S4-7**.


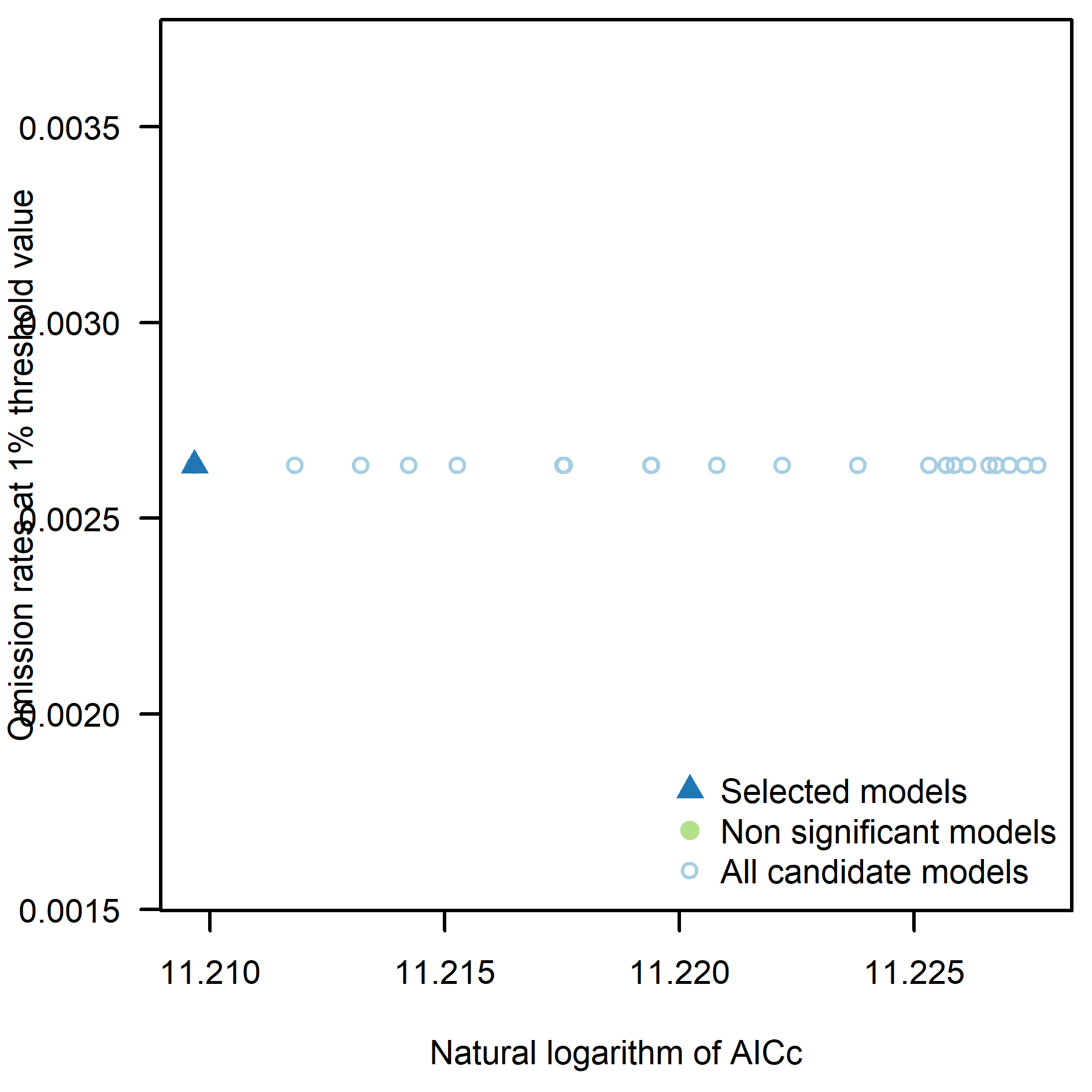


**Figure S4**. Model selection for *Salmo trutta fario*


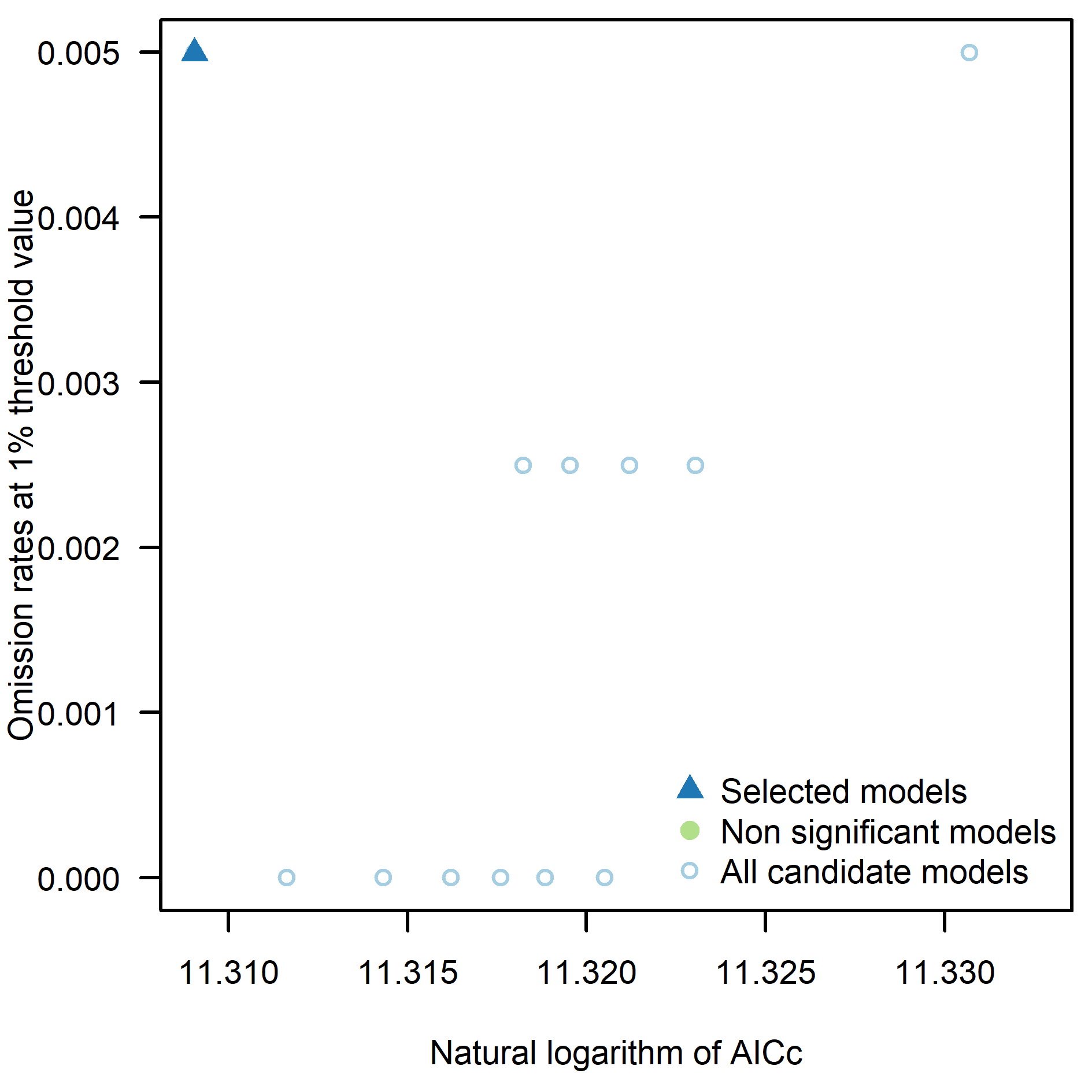


**Figure S5**. Model selection for *Oncorhynchus mykiss*


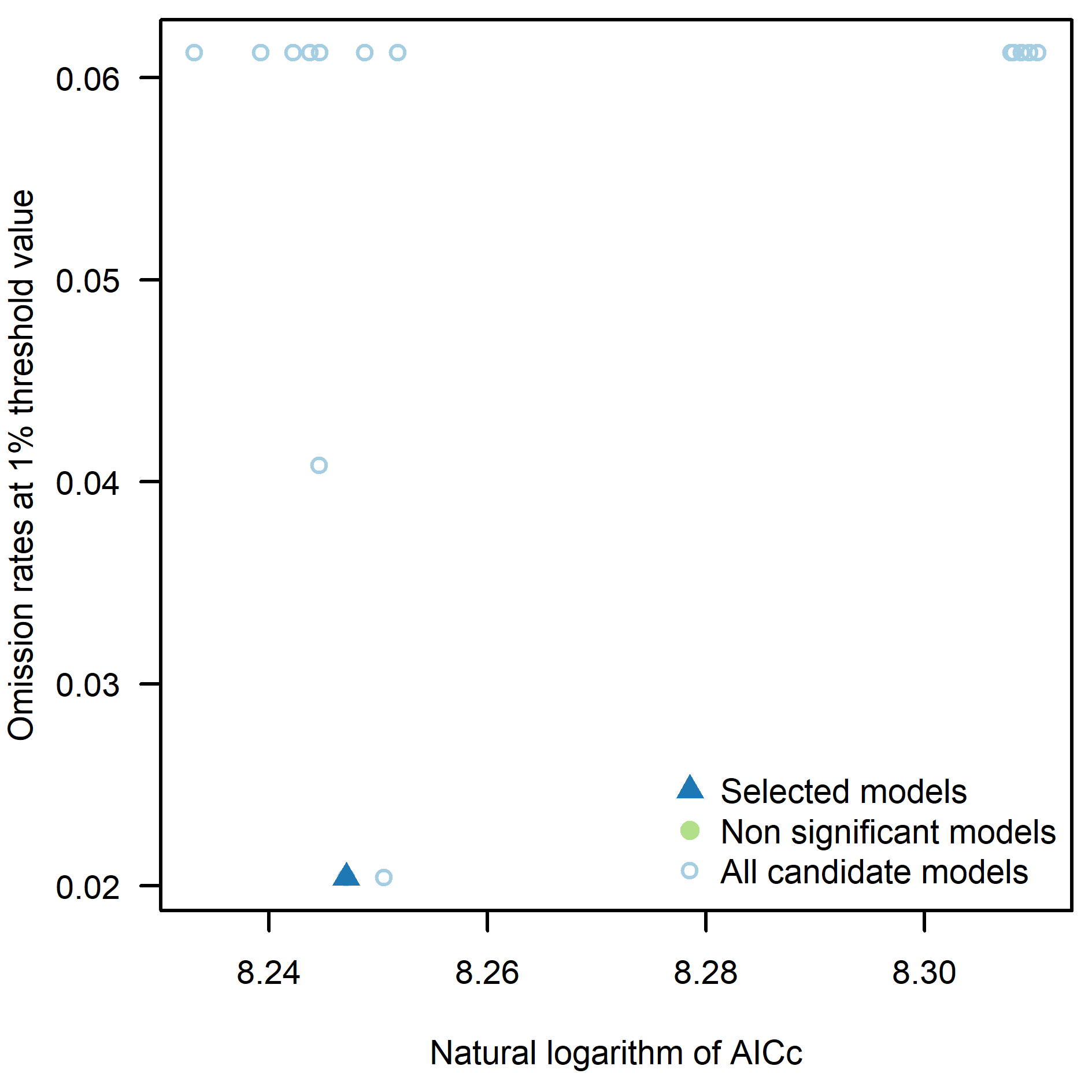


**Figure S6**. Model selection for *Schizothorax plagiostomus*


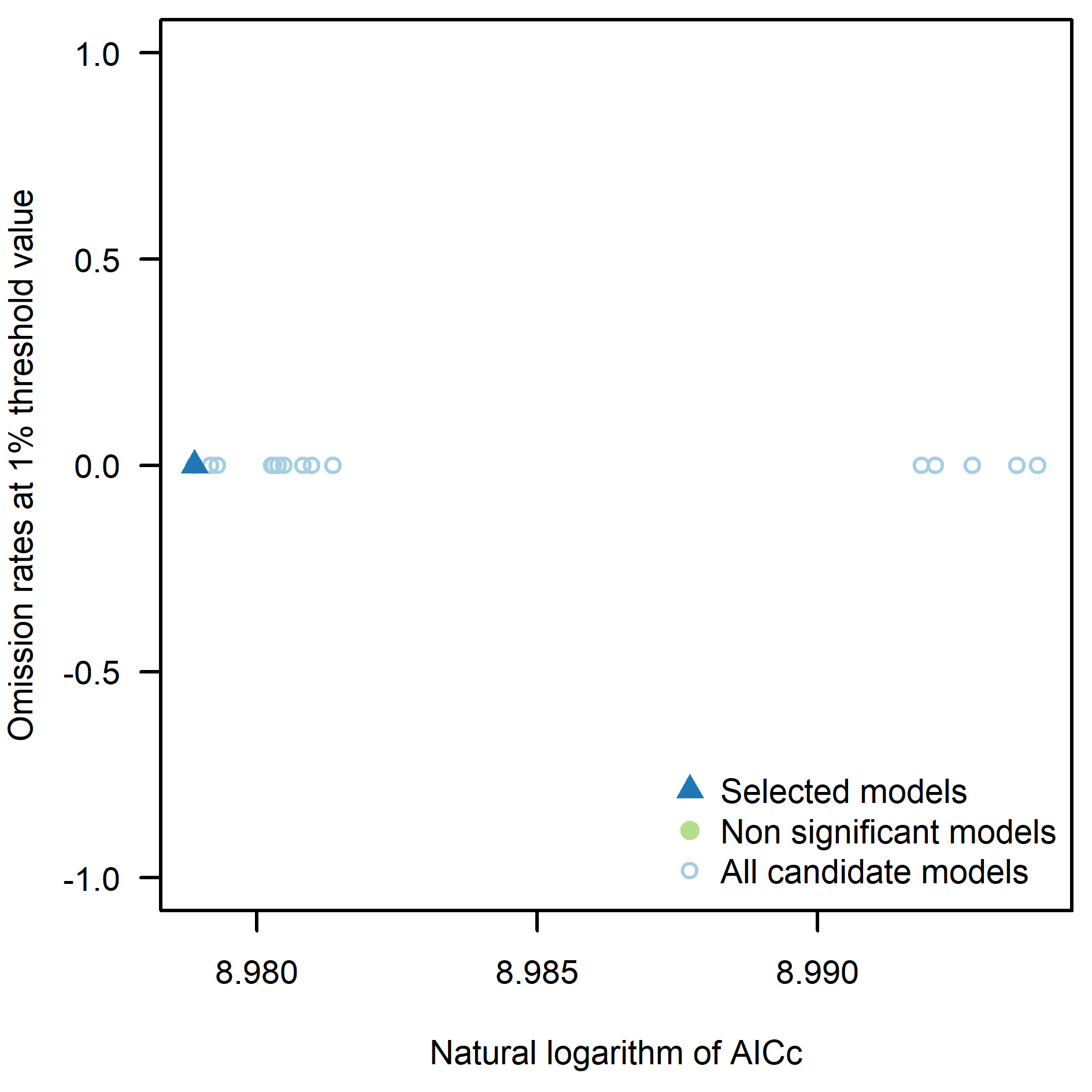


**Figure S7**. Model selection for *Schizothorax richardsonii*

1. **Selection of binary threshold**

MaxEnt models provide several options for selecting binary thresholds e.g., minimum training presence, equal sensitivity and specificity, and user specified arbitrary values based on omission rates. It has been argued that, in some cases, selecting appropriate thresholds are more important than presence locations ^7^. Threshold selection in this study was guided by the quality of occurrence data and study objectives. We have more confidence in data quality for *S. plagiostomus* as the lead author participated in the sampling efforts that accounted all occurrence locations of our study. Therefore, we used “minimum training presence” as binary threshold. For non-native trout species we selected the same thresholds i.e., “minimum training presence”. We were particularly cautious with the data cleaning process and discarded records of occurrences that had low confidence. In addition, the purpose of this study was to map all stream habitats that could potentially be invaded by these non-native species. Therefore, reasonable thresholds with minimum exclusion of such potentially suitable stream are best defined by “minimum training presence”. For non-native species, omission error would be more serious issue than commission error (i.e., not to find a non-native species in a habitat for which the model predict presence rather than the prediction of unsuitable habitats when it they are potentially suitable in reality). Collectively, for *S. plagiostomus* and non-native trout we used minimum training presence as binary thresholds whereas for *S. richardsonii*, some occurrence locations were obtained from Global biodiversity information facility (GBIF). We used “10 percentile training presence”, which leaves a 10% margin of error in the occurrence records and assumes that 10% of occurrence records in the least suitable habitat are not occurring in regions that are representative of the species overall habitat, and thus should be omitted.

1. **Evaluation of MaxEnt model through independent data**

As described in main text, using Receiver Operating Characteristic (ROC) analysis for model evaluation has been criticized for giving equal weight to omission and commission errors ^8^. Models for predicting suitable habitats for non-native/invasive species may have less tolerance for omission error than for commission error. Therefore, we used partial ROC (pROC) developed for ENM evaluation ^8^. pROC uses AUC ratios (The partial AUC divided by random expectation), where a value of 1.0 represents model performance no better than random whereas models with AUC ratios near 2.0 are considered good ^9^. The p-values of pROC indicate whether the ratios of model AUC to the random AUC is statistically significant.

| **Species** | **Validation data set** | | | **Independent data set** | | |
| --- | --- | --- | --- | --- | --- | --- |
|  | **Omission rate** | **Mean AUC ratio** | **Partial ROC** | **Omission rate** | **Mean AUC ratio** | **Partial ROC** |
| *S. plagiostomus* | 0.02 | 1.89 | 0.00 | 0.10 | 1.78 | 0.00 |
| *S. richardsonii* | 0.00 | 2.00 | 0.00 | No independent data available | | |
| *S. trutta* | 0.01 | 1.89 | 0.00 | 0.18 | 1.81 | 0.00 |
| *O. mykiss* | 0.01 | 1.99 | 0.00 | 0.07 | 1.97 | 0.00 |

**Table S1** Performance of models using pROC, omission rate and, AUC ratio for validation and independent datasets of species’ occurrences.

1. **Correlation plots**

**FigureS8**. Initial correlation plot with all candidate variables for *S. plagiostomus* and *S. richardsonii*. For both of these sympatric native snow trout species, one common background (range) was selected, hence one set of variables as shown in Table1 in the main article.


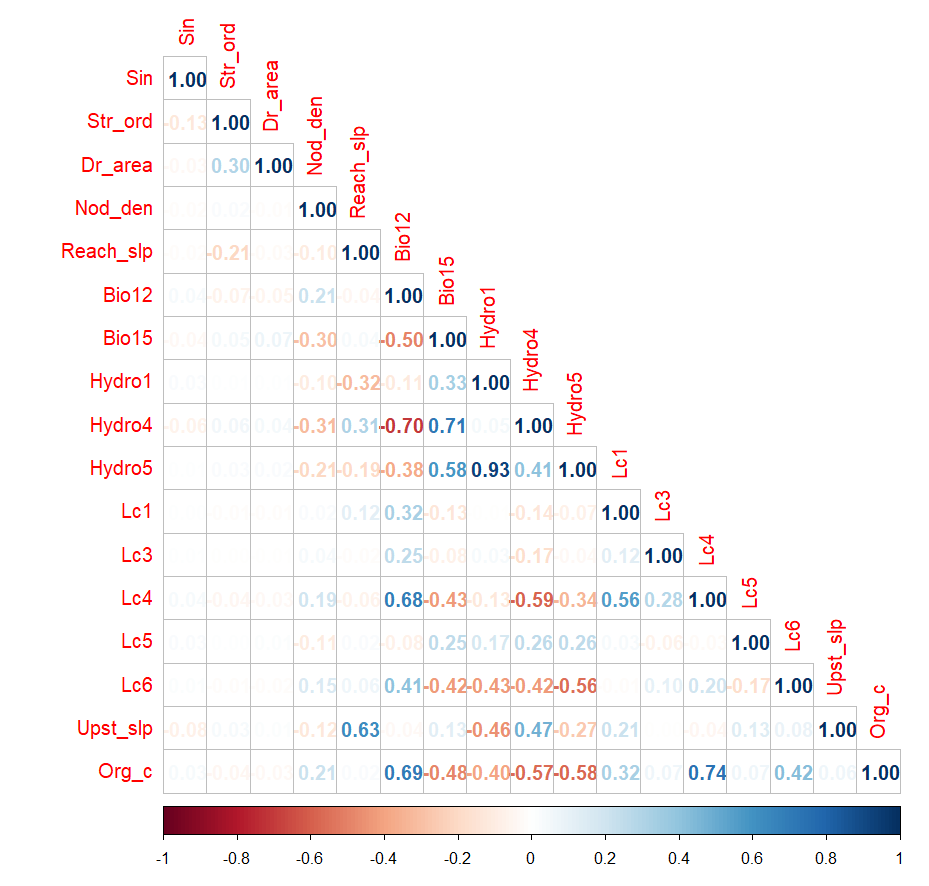


**Figure S9**. Final correlation plot for *S. plagiostomus* and *S. richardsonii* with highly correlated variable excluded.


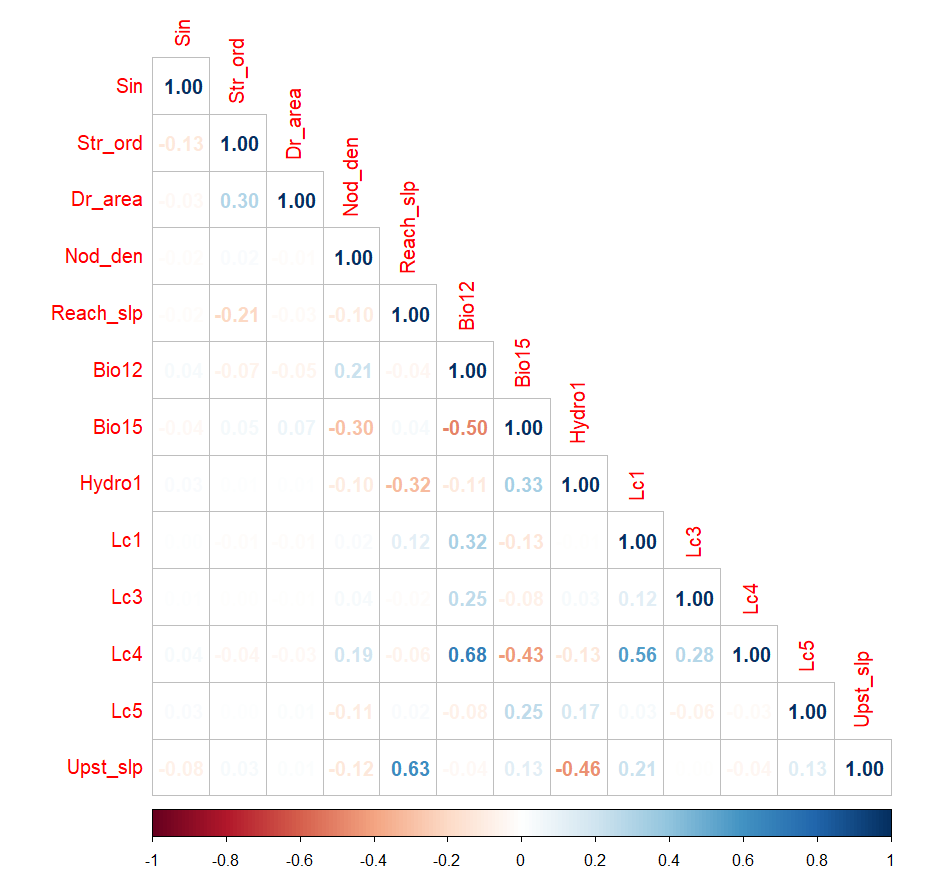


**Figure S10.** Initial correlation plot for Brown trout with all candidate variables.


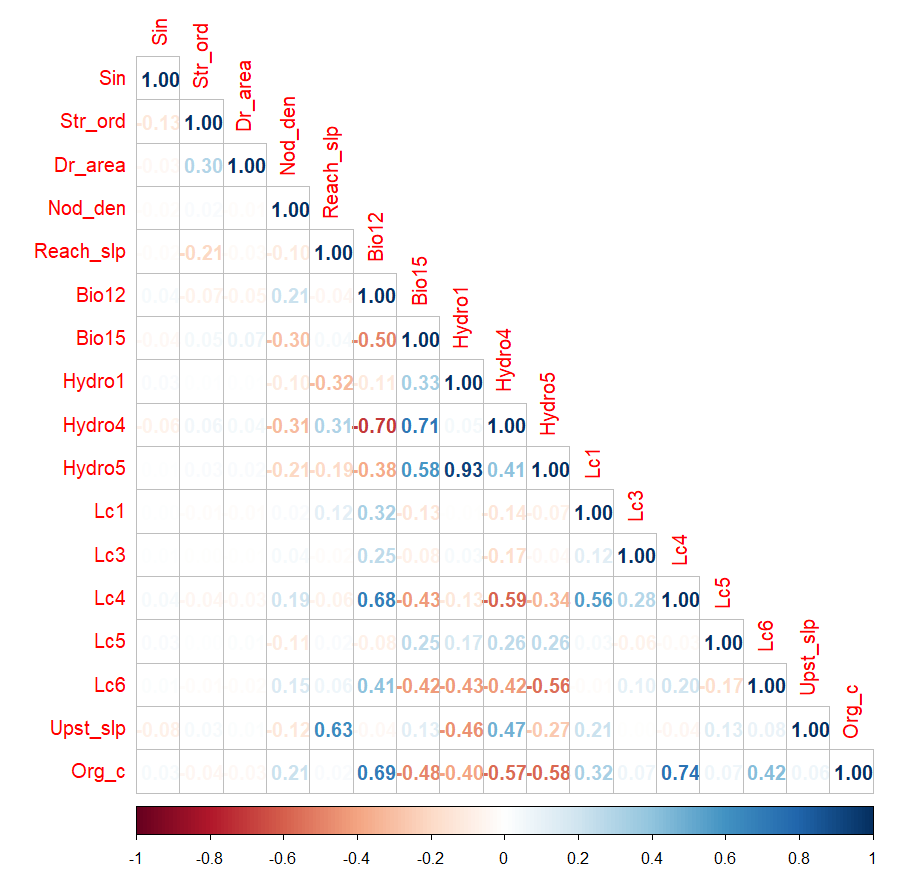


**Figure S11.** Final correlation plot for Brown trout with highly correlated variable excluded.


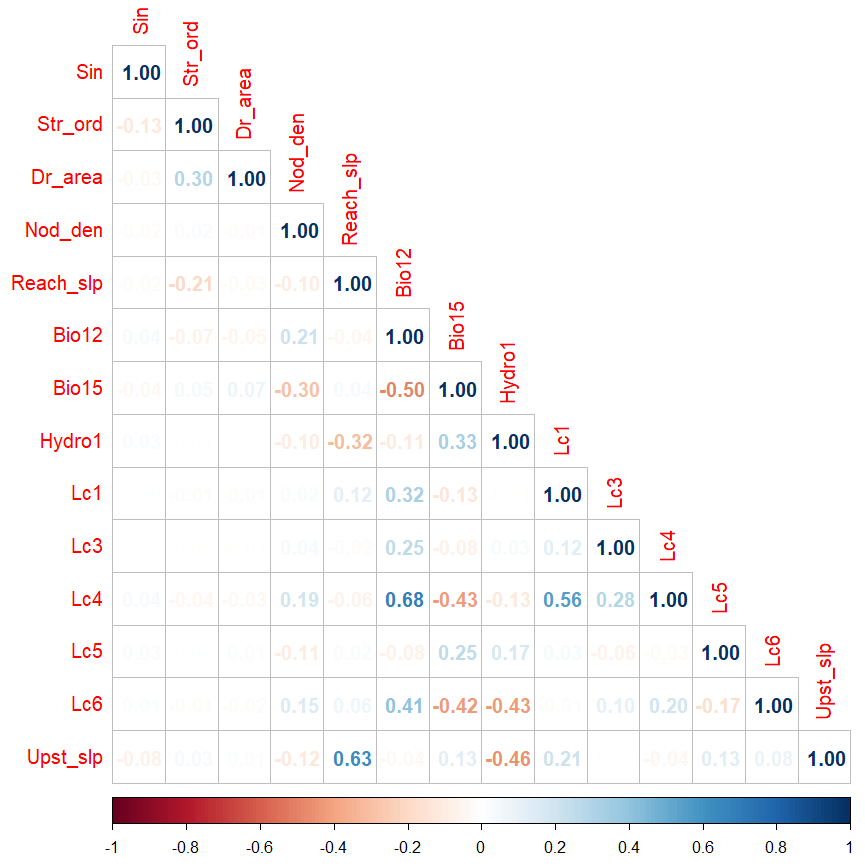


**Figure S12.** Initial correlation plot for Rainbow trout with all candidate variables.


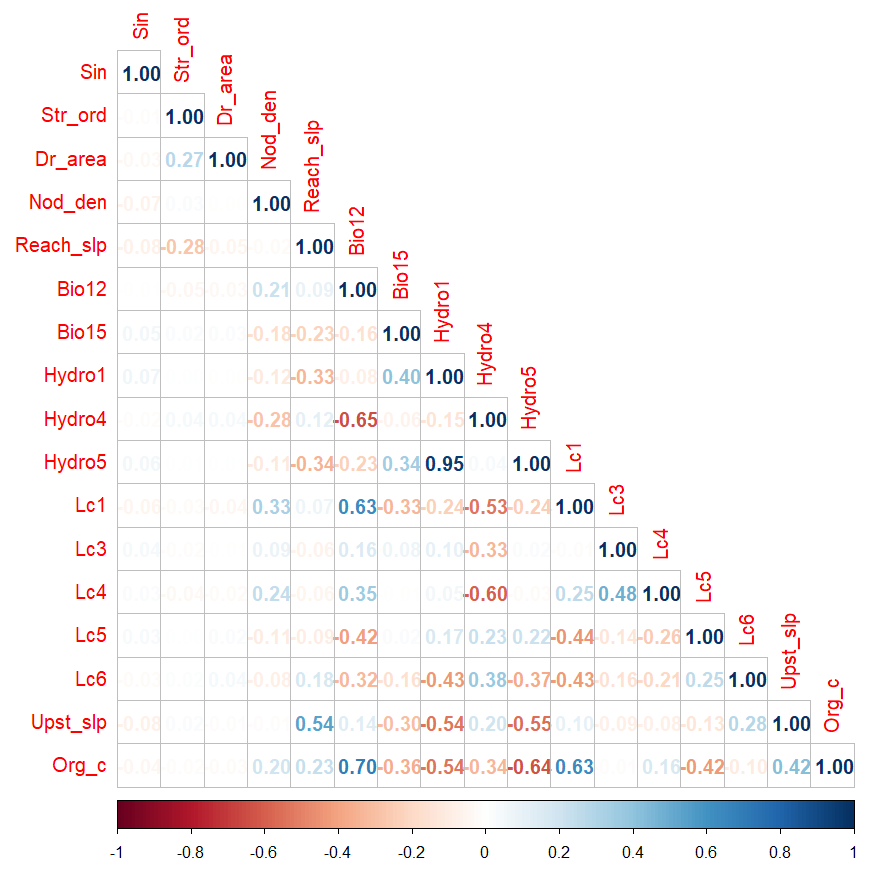


**Figure S13.** Final correlation plot for Rainbow trout with highly correlated variables excluded.


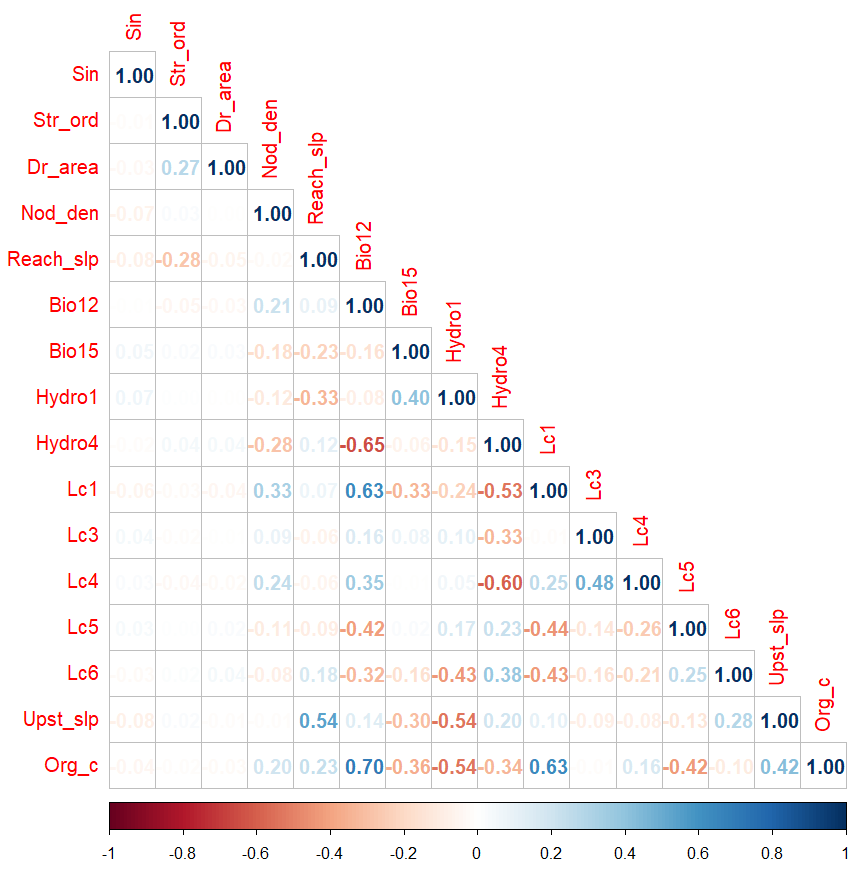


**Supplemental references**

[1]. Broennimann, O. *et al.* Evidence of climatic niche shift during biological invasion. *Ecology Letters* **10**, 701–709 (2007).

[2]. Brown, J. L. SDM toolbox: a python‐based GIS toolkit for landscape genetic, biogeographic and species distribution model analyses. *Methods in Ecology and Evolution* **5**, 694–700 (2014).

[3]. Peterson, A. T. *et al.* *Ecological niches and geographic distributions (MPB-49)*. (Princeton University Press, 2011).

[4]. Aiello‐Lammens, M. E., Boria, R. A., Radosavljevic, A., Vilela, B. & Anderson, R. P. spThin: an R package for spatial thinning of species occurrence records for use in ecological niche models. *Ecography* **38**, 541–545 (2015).

[5]. Renner, I. W. *et al.* Point process models for presence‐only analysis. *Methods in Ecology and Evolution* **6**, 366–379 (2015).

[6]. Cobos, M. E., Peterson, A. T., Barve, N. & Osorio-Olvera, L. kuenm: an R package for detailed development of ecological niche models using Maxent. *PeerJ* **7**, e6281 (2019).

[7]. Norris, D. Model Thresholds are More Important than Presence Location Type: Understanding the Distribution of Lowland tapir (Tapirus Terrestris) in a Continuous Atlantic Forest of Southeast Brazil. *Tropical Conservation Science* **7**, 529–547 (2014).

[8]. Peterson, A. T., Papeş, M. & Soberón, J. Rethinking receiver operating characteristic analysis applications in ecological niche modeling. *Ecological Modelling* **213**, 63–72 (2008).

[9]. Escobar, L. E., Qiao, H., Cabello, J. & Peterson, A. T. Ecological niche modeling re-examined: A case study with the Darwin’s fox. *Ecology and Evolution* **8**, 4757–4770 (2018).
